# Hederagenin from *Hedera helix* Promotes Fat Browning in 3T3-L1 Adipocytes

**DOI:** 10.3390/plants13192789

**Published:** 2024-10-04

**Authors:** Seung Min Choi, Ho Seon Lee, Sung Ho Lim, Gayoung Choi, Chang-Ik Choi

**Affiliations:** Integrated Research Institute for Drug Development, College of Pharmacy, Dongguk University-Seoul, Goyang 10326, Republic of Korea; tlsehdhs@dgu.ac.kr (S.M.C.); ghtjsrhtn@dongguk.edu (H.S.L.); sho617@dgu.ac.kr (S.H.L.); gayoung@dgu.ac.kr (G.C.)

**Keywords:** hederagenin, fat browning, *Hedera helix*, 3T3-L1, uncoupling protein 1

## Abstract

The prevalence of obesity is increasing globally, with approximately 700 million obese people worldwide. Currently, regulating energy homeostasis by increasing energy expenditure is attracting attention as a strategy for treating obesity. White adipose tissue is known to play a role in accumulating energy by storing excess energy, while brown adipose tissue expends energy and maintains body temperature. Thus, the browning of white adipose tissue has been shown to be effective in controlling obesity. *Hedera helix* (*H. helix*) has been widely used as a traditional medicine for various diseases. In several previous studies, hederagenin (HDG) from *H. helix* has demonstrated many biological activities. In this study, we investigated the antiobesity effect of HDG on fat browning in 3T3-L1 adipocytes. Consequent to HDG treatment, a reduction in lipid accumulation was measured through oil red O staining. In addition, this study investigated that HDG increases energy expenditure by upregulating the expression of several targets related to thermogenesis, including uncoupling protein 1 (UCP1). This process involves inhibiting lipogenesis via the adenosine monophosphate-activated protein kinase (AMPK) signaling pathway and promoting lipolysis through the protein kinase A (PKA) pathway. HDG is expected to be effective in promoting fat browning, indicating its potential as a natural antiobesity candidate.

## 1. Introduction

The prevalence of obesity is rising globally, with approximately 2.2 billion people overweight and 700 million people obese [1]. Obesity causes adverse health effects and various metabolic complications, including dyslipidemia, insulin resistance, type 2 diabetes, fatty liver, and cardiovascular disease [2]. Additionally, obesity is associated with a higher incidence of cancer [3]. Various methods to combat obesity have been explored [4], such as exercise, diet, and bariatric surgery. However, diet and exercise alone are often only partially successful [5]. Bariatric surgery is typically reserved for selected cases. Hence, developing drugs to treat obesity that can replace or supplement methods such as diet, exercise, and surgical treatment is necessary.

Three main pharmacological approaches exist for treating obesity: decreasing energy intake through appetite suppression, reducing energy absorption owing to malabsorption, and increasing energy expenditure [6]. Among these, strategies to increase energy expenditure are particularly significant because, unlike appetite suppression or energy intake reduction, they can be achieved without affecting the absorption of essential nutrients such as vitamins and minerals [4].

Mammals have two types of adipose tissue: white adipose tissue (WAT) and brown adipose tissue (BAT). WAT stores excess energy in the form of triglycerides, while BAT maintains core body temperature via energy expenditure [1]. In humans, a large amount of brown fat is present during infancy [7] but gradually decreases with age [8]. For a long time, brown fat was originally believed to not exist in adult humans, but later, studies discovered substantial amounts of brown and beige fat cells in the neck and shoulder areas of adults, confirming its presence [9]. Additionally, Cypess et al. investigated the presence of brown fat in adult humans when exposed to cold [10]. The activation of BAT and browning of WAT accelerates glycolipid intake, reduces the requirement for insulin secretion, and improves glycolipid metabolism and insulin resistance in patients with obesity and type 2 diabetes [11,12,13]. The thermogenic activity of BAT primarily relies on uncoupling protein 1 (UCP1), located in the inner mitochondrial membrane. When activated, UCP1 catalyzes the leak of protons across the mitochondrial membrane, uncoupling oxidative respiration from ATP synthesis and releasing energy as heat. Beige adipocytes, formed through fat browning, are also rich in the mitochondria and UCP1, increasing energy expenditure in response to cold and various stimuli [14]. Numerous studies are currently exploring the effectiveness of ingredients derived from natural products in promoting fat browning [15,16,17,18,19]. Therefore, WAT browning is gaining attention as a promising strategy for treating obesity by increasing energy expenditure.

**Figure 1:**
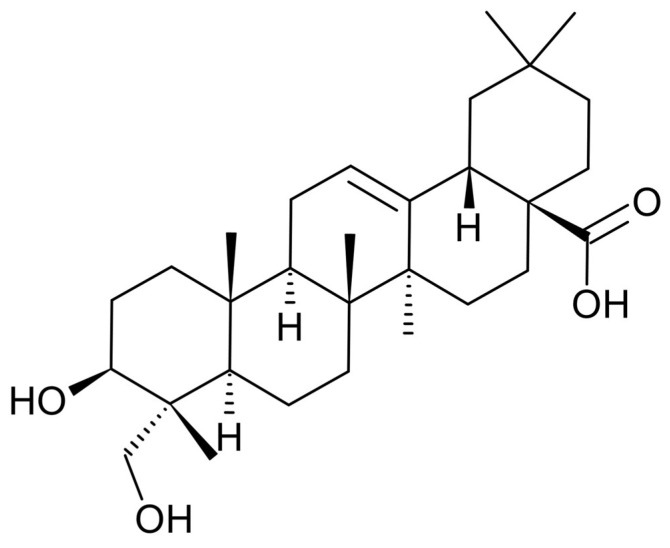
Chemical structure of hederagenin (HDG).

*Hedera helix* (*H. helix*), commonly known as ivy, contains various chemical components, including triterpene saponins, flavonoids, polyacetylenes, and phenolic compounds [20,21]. Traditionally used to treat respiratory diseases since the 19th century [22], *H. helix* has become standardized in modern medicine, with its extracts used to manufacture various types of medicines, such as syrups, suppositories, and eye drops [23]. Studies show that *H. helix* exhibits various bioactivities, including anti-inflammatory, analgesic, antimicrobial, antioxidant, anticancer, and antidiabetic effects, alongside its effects on respiratory diseases [24,25,26,27,28,29]. One key compound in *H. helix* is hederagenin (HDG) (Figure 1), a triterpene that serves as an indicator component of the plant. HDG has been extensively examined for its bioactivity, including anti-inflammatory, antifungal, antibacterial, antidiabetic, antidepressant, antineurodegenerative, antitumor, and antiatherosclerosis effects [30]. However, research on the antiobesity effect of HDG, particularly related to fat browning, is lacking. Therefore, this study aims to explore the effect of HDG on fat browning and its underlying mechanism in 3T3-L1 adipocytes.

## 2. Results

### 2.1. Effect of HDG on Cell Viability

**Figure 2:**
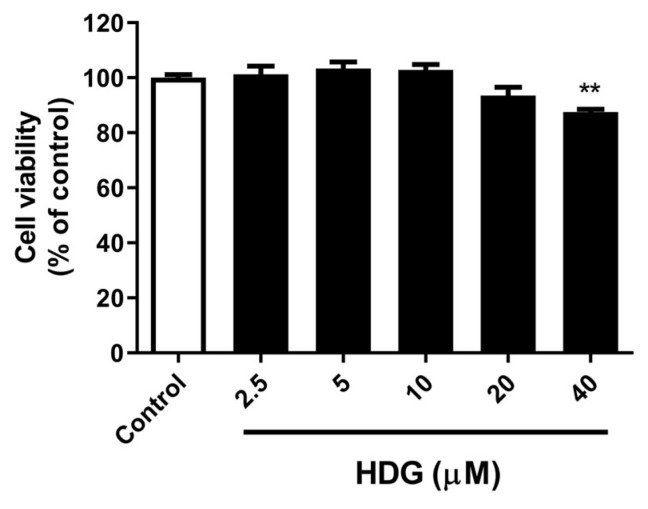
Effects of HDG on cell viability of 3T3-L1 preadipocytes. The cells were treated with the compound and incubated for 48 h. All data are presented as mean ± SEM (n = 3). ** *p* < 0.01 compared with the control. Statistical analysis was conducted using Student’s *t*-test.

The effect of HDG on cell viability was examined in 3T3-L1 cells using a thiazolyl blue tetrazolium bromide (MTT) assay. HDG was administered at concentrations ranging from 2.5 to 40 μM for up to 48 h. Cell viability showed a slight reduction at the highest concentration of 40 μM. In subsequent experiments, the maximum concentration was set to 20 μM, which did not significantly affect cell viability (Figure 2).

### 2.2. Effect of HDG on Lipid Accumulation

**Figure 3:**
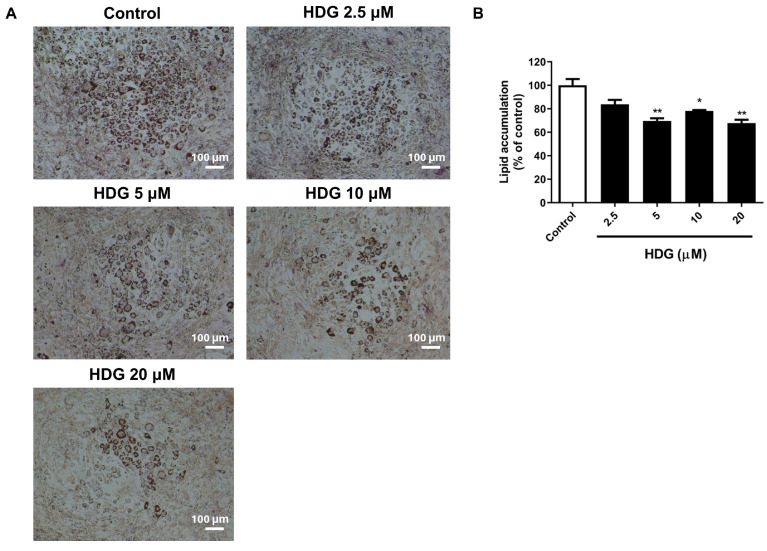
Effects of HDG on lipid accumulation in 3T3-L1 cells. The cells were differentiated for 7 days and stained with ORO (magnification: ×100, scale bar = 100 µm) (**A**). The stained cells were eluted with 100% isopropanol. Lipid accumulation was quantified using a microplate reader at 520 nm (**B**). All data are presented as mean ± SEM (n = 3). * *p* < 0.05 or ** *p* < 0.01 compared with the control. Statistical analysis was performed using Student’s *t*-test.

Lipid accumulation was evaluated using oil red O (ORO) staining. A decrease in lipid accumulation was observed at concentrations of 5, 10, and 20 μM (Figure 3A), with the lowest level occurring at 20 μM (Figure 3B). These results prompted further investigation into lipid metabolism related to fat browning.

### 2.3. Effect of HDG on Mitochondrial Biogenesis and Adipocyte Browning

**Figure 4:**
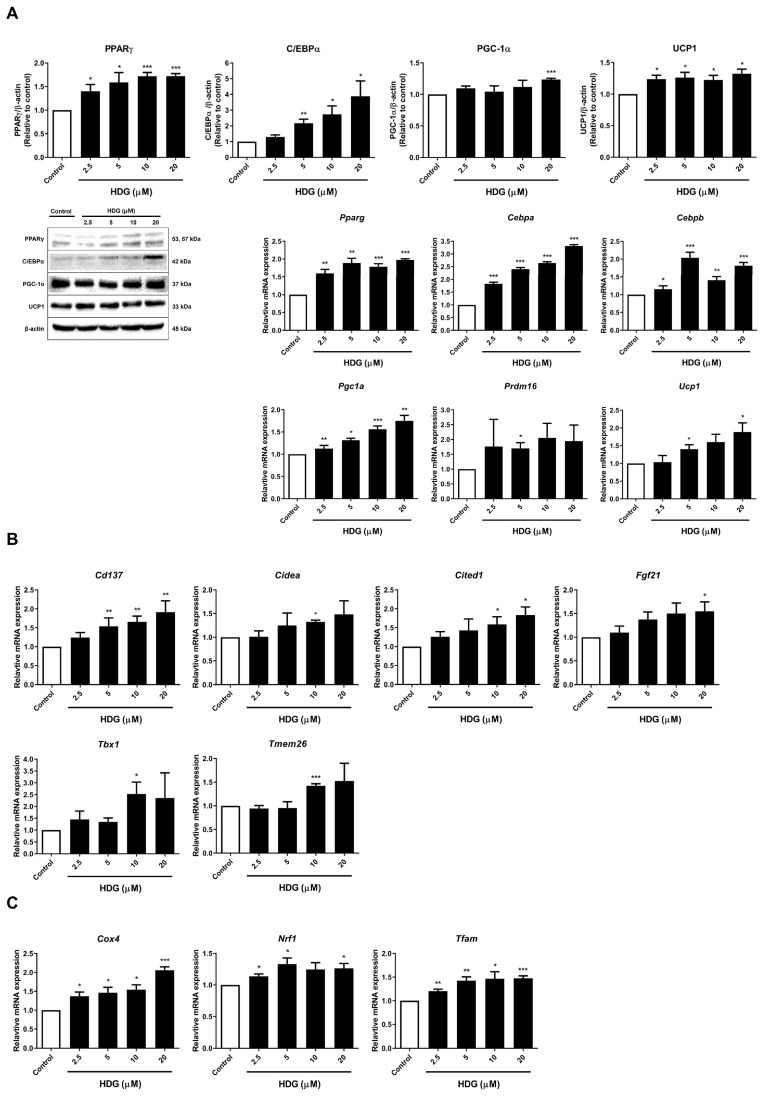
Effects of HDG on the expression of thermogenesis markers (**A**), beige-specific markers (**B**), and mitochondrial biogenesis markers (**C**). All data are presented as mean ± SEM (n = 3). * *p* < 0.05, ** *p* < 0.01, and *** *p* < 0.001 compared with the control. Statistical analysis was conducted using Student’s *t*-test.

To assess the thermogenic activity of HDG in *H. helix*, we identified markers such as UCP1 and peroxisome proliferator activated-receptor gamma coactivator-1 alpha (PGC-1α). We also assessed the protein levels of peroxisome proliferator activated-receptor gamma (PPARγ) and CCAAT/enhancer-binding protein alpha (C/EBPα) alongside the mRNA expression levels of *Pparg*, *Cebpa*, and CCAAT/enhancer-binding protein beta (*Cebpb*). These factors collaborate with PGC-1α to promote the transcriptional expression of UCP1. HDG treatment induced the highest expression of all fat-browning-specific markers, except for *Cebpb* and the PR domain containing 16 (*Prdm16*), at the highest concentration of 20 μM. At 5 μM, *Cebpb* and *Prdm16* exhibited the highest expression levels, which were statistically significant (Figure 4A).

Beige-specific markers, indicative of increased expression in beige adipocytes during fat browning, were investigated in this study. We analyzed the mRNA expression of six targets: tumor necrosis factor receptor superfamily, member 9 (*TNFRSF9*, *Cd137*), cell death inducing DFFA-like effector A (*Cidea*), carboxy-terminal domain 1 (*Cited1*), fibroblast growth factor 21 (*Fgf21*), T-box 1 (*Tbx1*), and transmembrane protein 26 (*Tmem26*). Treatment with HDG resulted in the highest increase in expression for all targets at 10 or 20 μM (Figure 4B). These results indicate that HDG treatment promotes fat browning as the beige-specific marker increased. Additionally, given that the activation of fat browning likely affects mitochondria, where UCP1 is located, we measured the mRNA expression of three mitochondrial biogenesis markers: cytochrome c oxidase subunit 4 (*Cox4*), nuclear respiratory factor 1 (*Nrf1*), and mitochondrial transcription factor A (*Tfam*). HDG treatment elevated the expression levels of these markers. At the highest HDG concentration, the expression levels of *Cox4* were approximately 2-fold higher, and *Tfam* was approximately 1.5-fold higher. *Nrf1* exhibited the highest expression level at 5 μM, increasing by approximately 1.3 times compared to that of the control (Figure 4C).

**Figure 5:**
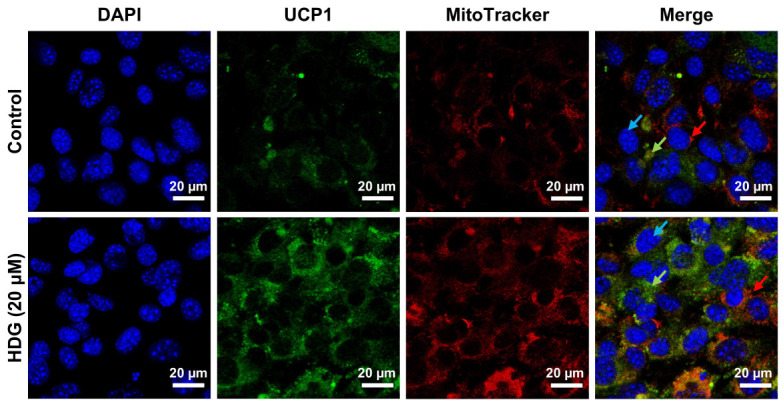
Effect of HDG on UCP1 and mitochondria measured using immunofluorescence staining. 3T3-L1 adipocytes treated with 20 μM HDG for 7 days were stained for UCP1-FITC, MitoTracker Red, and DAPI. The images were captured at 60× magnification (scale bars = 20 µm). The blue, red, and green arrows indicate the adipocyte nucleus, mitochondria, and UCP1, respectively.

Consistent with this, UCP1 showed increased expression at the highest concentration when measured using immunofluorescence. Furthermore, MitoTracker Red displayed an elevated expression compared to that of the control and a similar expression pattern to that of UCP1 (Figure 5). These results demonstrate that HDG affects mitochondrial biogenesis. Collectively, these findings suggest that HDG enhances markers of fat browning and thermogenesis.

### 2.4. Effect of HDG on Lipogenesis and Lipolysis

**Figure 6:**
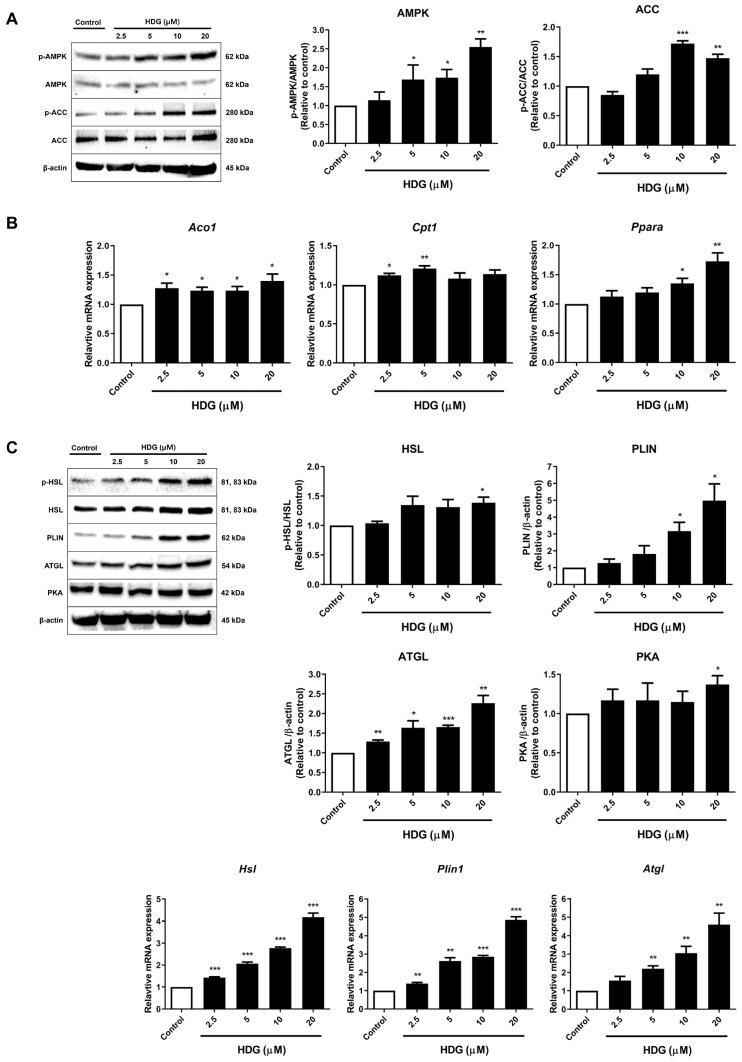
Effects of HDG on the expression of lipogenesis pathway proteins (**A**), fatty acid oxidation markers (**B**), and lipolysis markers (**C**). All data are presented as mean ± SEM (n = 3). * *p* < 0.05, ** *p* < 0.01, and *** *p* < 0.001 compared with the control. Statistical analysis was conducted using Student’s *t*-test.

We explored the effects of HDG on lipogenesis markers acetyl CoA carboxylase (ACC) and adenosine monophosphate-activated protein kinase (AMPK). The results showed an increase in the expression level of p-AMPK, which peaked at the highest HDG concentration of 20 μM (Figure 6A). ACC exhibited the highest phosphorylation level at a concentration of 10 μM (Figure 6A). This suggests that HDG may inhibit lipogenesis in 3T3-L1 cells. Fatty acid oxidation, which breaks down fats, is regulated by the phosphorylation of AMPK and ACC. The p-AMPK enhances the expression of carnitine palmitoyl transferase 1 (*Cpt1*, a fatty acid oxidation marker), thereby promoting fatty acid oxidation, which provides the protons necessary for the activation of UCP1 and subsequent thermogenesis. In this study, we assessed the mRNA expression of three fatty acid oxidation markers: acyl-CoA oxidase 1 (*Aco1*), *Cpt1*, and peroxisome proliferator activated-receptor alpha (*Ppara*). HDG treatment resulted in an increase in the expression levels of *Aco1* and *Ppara* at the highest concentration (Figure 6B), indicating that HDG influences fatty acid oxidation. Lipolysis provides the energy required for thermogenesis. Therefore, we investigated the expression of lipolysis-related markers in fat browning. HDG treatment led to increased expression levels of phosphorylated hormone-sensitive lipase (HSL), perilipin (PLIN), adipose triglyceride lipase (ATGL), and protein kinase A (PKA). Particularly, ATGL increased in a dose-dependent manner (Figure 6C). Moreover, the mRNA expression levels of *Hsl* and *Plin* showed concentration-dependent increases (Figure 6C). These results show that HDG has lipolytic activity.

## 3. Discussion

As obesity continues to pose a significant global health threat [1], various methods are being explored to combat it. Among these, fat browning, which transforms WAT into BAT and increases energy expenditure, has emerged as a promising strategy [4]. Previous studies showed that *H. helix* exhibits bioactive effects, including anti-inflammatory, antioxidant, anticancer, and antidiabetic effects [24,25,26,27,28,29]. Given the close relationship between these diseases and obesity, investigating the effect of *H. helix* on obesity is warranted. In this study, we assessed the fat-browning effect of HDG in *H. helix* and found that the compound promotes the browning of white adipocytes, converting them into beige adipocytes.

The crucial factor in fat browning is UCP1, a key protein in thermogenesis that utilizes excess energy to maintain thermogenesis and energy balance [31,32]. The ectopic expression of UCP1 and brown-adipocyte-characteristic proteins, such as PGC-1α and PRDM16, in white adipocytes signify the transition from WAT to BAT [33,34]. PGC-1α links the nucleus and mitochondria, boosting mitochondrial biogenesis and activating PPARγ. When PGC-1α activates TFAM, an increased expression of COX4 and activation of NRF1 can be observed, which triggers mitochondrial replication [31]. Mitochondria are essential organelles involved in energy metabolism and play a critical role in the thermogenesis of adipocytes. An increase in both the number and activity of mitochondria is a hallmark of the browning process. Mitochondrial dysfunction in adipocytes can adversely impact lipid metabolism, insulin sensitivity, and thermogenesis, contributing to metabolic diseases such as obesity and type 2 diabetes [18]. Our results demonstrated an increase in PGC-1α, a key regulator of mitochondrial biogenesis, along with important regulators *Cox4*, *Nrf1*, and *Tfam*, which interact with PGC-1α. Given that mitochondrial dysfunction is a significant risk factor for the development of obesity and diabetes, enhancing mitochondrial biogenesis through HDG is expected to be effective in addressing various metabolic syndromes. *Prdm16*, a common gene in BAT, increases during fat browning [35]. The expression of this marker is known to decrease when beige adipocytes are converted back to white adipocytes, suggesting that PRDM16 plays a central role in fat browning and the maintenance of beige adipocytes. Additionally, PGC-1α and PRDM16 form a transcription complex with PPARγ, C/EBPα, and C/EBPβ, which are ultimately involved in UCP1 expression. Considering that UCP1, located in the mitochondrial inner membrane, is crucial in thermogenesis, PPARγ, C/EBPα, and C/EBPβ, along with PGC-1α, play pivotal roles in this process [36]. The results of the present study showed that HDG increased UCP1 expression at the highest concentration. HDG generally enhanced thermogenesis and elevated the expression of thermogenesis-related markers.

AMPK is a key regulator of metabolism, promoting energy-generating pathways while inhibiting energy-storage pathways [37]. Numerous studies have shown that when activated, AMPK is associated with fatty acid oxidation, BAT thermogenesis, and WAT browning, including lipogenesis. AMPK is a highly conserved and ubiquitously expressed serine/threonine protein kinase and has a multiheterotrimeric complex structure composed of α, β, and γ subunits. The α catalytic subunit includes an N-terminal kinase domain, an auto-inhibitory domain, and a C-terminal β/γ subunit-binding domain. AMPK is activated when the Thr-172 residue, the catalytic phosphorylation site, is phosphorylated [38]. Additionally, activated AMPK participates in mitochondrial fatty acid oxidation and phosphorylates ACC, an inhibitor of CPT1 activity, thus halting fatty acid synthesis [39]. AMPK also upregulates PGC-1α and PRDM16. Through this, elevated PPARα is known to play a role in fatty acid oxidation, together with CPT1 [40,41]. ACO1 is an enzyme involved in the initial step of fatty acid oxidation [42]. When these targets promote fatty acid oxidation, the oxidized fatty acids work with UCP1 in the mitochondria to enhance thermogenesis. Our results showed that HDG treatment increased the phosphorylation levels of AMPK and ACC, suggesting that thermogenesis is enhanced through the phosphorylation of AMPK.

The mobilization of metabolic energy from adipocytes relies on a tightly regulated balance between the hydrolysis and resynthesis of triglycerides [43]. The hydrolysis of triglycerides activates the cAMP-PKA pathway via β-adrenergic signaling, leading to the phosphorylation and activation of lipolytic enzymes, including HSL, ATGL, and PLIN [44,45]. HSL phosphorylation occurs at several sites, including ser-563, and HSL phosphorylated by PKA affects lipolysis in adipocytes to maintain whole-body energy homeostasis [43]. In brown and beige adipocytes, fatty acids activate UCP1, enabling maximum mitochondrial oxidation rates without ATP synthesis, which fuels high thermogenesis. Therefore, the mobilization of free fatty acids through lipolysis in brown and beige adipocytes is crucial for thermogenesis [46]. Pharmacological inhibition of the two-step catalytic proteins ATGL and HSL, which are responsible for triglyceride hydrolysis, has been shown to reduce the adrenergic stimulation of thermogenesis completely. In addition, Li et al. found that the addition of free fatty acids stimulates thermogenesis in brown adipocytes even in the absence of adrenergic stimulation [47]. In our experiment, similar to the results for thermogenesis markers, the highest concentration of HDG showed a strong lipolysis effect, significantly increasing all protein and mRNA targets. 

Consistent with our study, similar trends were observed in fat browning research involving various natural compounds and extracts using 3T3-L1 cells. A study by Liu et al. [16] using peanut shell extract showed an increase in p-ACC, p-AMPK, p-HSL, PGC-1α, *Tfam*, *Nrf1*, and UCP1. The results in Mulberry leaf flavonoids indicated an increase in p-AMPK, *Cd137*, CPT-1, PGC-1α, PRDM16, *Tbx1*, *Tmem26*, and UCP1 [18]. Zeaxanthin [48] demonstrated an upregulation of *Nrf1*, PGC-1α, PKA, PRDM16, *Tfam*, and UCP1. A study on Black Ginseng and Ginsenoside Rb1 showed an increase in p-AMPK, PGC-1α, PRDM16, and UCP1 [49]. These results strongly support that the upregulation of these targets by hederagenin may positively influence fat browning through the AMPK and PKA signaling pathways. Some studies utilized inhibitors and activators of AMPK and PKA to validate the expected effects of inhibition and activation of the corresponding signaling pathways [48,49]. These results represent effective approaches for evaluating signaling pathways. Using inhibitors and activators of AMPK and PKA can yield substantial data to substantiate the signaling pathways involved in the fat browning effect of HDG.

## 4. Materials and Methods

### 4.1. Chemical Reagents

3T3-L1 white preadipocytes were obtained from the Korea Cell Line Bank (Seoul, Korea). HDG (purity ≥ 98%) was sourced from ChemFaces Biochemical Co. (Hubei, China). Dexamethasone, insulin, phosphate-buffered saline (PBS), ORO, and 10% formalin were procured from Sigma-Aldrich (St. Louis, MO, USA). Secondary antibodies (goat anti-rabbit IgG, goat anti-mouse IgG), 3-isobutyl-1-methylxanthine, and isopropanol were purchased from Merck (Union County, NJ, USA). Dulbecco’s Modified Eagle’s Medium (high glucose) (DMEM), newborn bovine calf serum (NCS), fetal bovine serum, 4′,6-diamidino-2-phenylindole (DAPI), and penicillin–streptomycin solution were purchased from Thermo Fisher Scientific (Waltham, MA, USA). Dimethyl sulfoxide (DMSO) and MTT were sourced from GlenthamLife Sciences (Corsham, UK). MitoTracker^®^ Red CMXRos was obtained from Cell Signaling Technology (Danvers, MA, USA). Triton^®^ X-100 was obtained from Promega (Madison, WI, USA). Antibodies used in the Western blot—PPARγ (2435S), C/EBPα (8178S), AMPK (2532S), phosphorylated AMPK (p-AMPK, 2531S), ACC (3662S), phosphorylated ACC (p-ACC, 3661S), HSL (18381S), phosphorylated HSL (p-HSL, 4139S), ATGL (2439S), PLIN (9349S), PKA (5842S), and β-actin (4967S)—were procured from Cell Signaling Technology (Danvers, MA, USA). UCP1 (sc-293418), UCP1-Fluorescein isothiocyanate (sc-518171 FITC), and PGC-1α (sc-518025) were acquired from Santa Cruz Biotechnology (CA, USA). For quantitative real-time polymerase chain reaction (qRT-PCR), NucleoZOL and the NucleoSpin^®^ RNA kit were obtained from MACHEREY-NAGEL (Düren, Germany). The ReverTra Ace^®^qPCR RT kit was purchased from TOYOBO (Osaka, Japan).

### 4.2. Cell Culture and Differentiation

3T3-L1 preadipocytes were cultured in DMEM with NCS for 3 days, subcultured, and then differentiated (37 °C in a 5% CO_2_ incubator). Once the cells were fully grown, the medium was replaced with differentiation initiation medium and cultured for 7 days. During the differentiation process, HDG, dissolved in DMSO at concentrations of 2.5 to 20 μM, was added. 

### 4.3. Cell Viability Assay

The medium was then replaced and treated with HDG at the specified concentration for 48 h. After treatment, 20 μL of MTT solution (5 mg/mL in PBS) was added to each well and incubated in a CO_2_ incubator for 2 h. After the reaction was completed, the medium and MTT solution were removed, and 100 μL of DMSO was added to dissolve the formazan crystals under dark conditions for 10 min. Cell viability was determined by measuring the absorbance value at 540 nm using a spectrophotometer.

### 4.4. Oil Red O Staining

The cells were fixed with 10% formalin and washed twice with distilled water (DW). The ORO solution and DW were mixed in a 6:4 ratio, added to each well (500 μL/well), and incubated at 25 °C for 30 min. Photographs were taken under a microscope. The 24-well plate was inverted and dried in dark conditions at 25 °C for 24 h to ensure complete drying. Lipid accumulation was assessed by measuring the absorbance at 520 nm using a spectrophotometer.

### 4.5. Mitochondrial Analysis and Immunofluorescence

3T3-L1 preadipocytes were cultured (1 × 10^5^ cells/well) and differentiated in 24-well plates using sterile coverslips. After differentiation, MitoTracker^®^ Red CMXRos (50 nM) was incorporated into the growth medium for mitochondrial staining and incubated for 30 min. The cells were fixed with 10% formalin for 15 min at 25 °C. Following fixation, the cells were treated with a blocking buffer (5% bovine serum albumin, 0.1% Triton^®^). The primary antibody (UCP1, diluted 1:250 in the blocking buffer) was incubated overnight at 4 °C. Subsequently, the cells were treated with DAPI (1:2000) for 1 min for nuclear staining. A coverslip containing the stained cells was placed on a glass slide to dry. Fluorescence images were captured using EZ-C1 software version 3.9 (Nikon, Tokyo, Japan) on a Nikon C1 confocal laser scanning microscopy apparatus.

### 4.6. Western Blot Analysis

The differentiated 3T3-L1 cells (1 × 10^6^ cells/well) were scraped and centrifuged (14,000 rpm, 5 min). After removing the resulting supernatant, lysis buffer (Radio-immunoprecipitation assay buffer 98%, a phosphatase inhibitor 1%, and a protease inhibitor 1%) was added to the cell pellet, mixed, and then incubated on ice for 30 min. The mixture was then centrifuged, and the resulting supernatant was used in the next step. The quantified protein (40 μg) was prepared by adding DW and sample buffer (900 μL of 4 × Laemmli sample buffer + 100 μL of 2-mercaptoethanol). Samples were boiled at 100 °C for 10 min and then centrifuged (4 °C, 15,000 rpm). Depending on the protein target, a 10% or 15% gel was prepared and placed in a PowerPac for electrophoresis (80 V, 20 min; 120 V, 60 min). Electrophoresed protein samples were transferred to an Immun-Blot^®^ PVDF membrane for 1 h (100 V, 60 min). Blocking was performed by shaking the membrane for 1 h in 5% skim milk diluted with Tris buffer saline (TBS) with Tween 20 (TBS-T) (DW 900 mL + 10× TBS buffer 100 mL + 10% Tween 20 solution 1 mL) buffer. The membrane was shaken with the primary antibody (diluted 1:1000, in TBS-T containing 5% bovine serum albumin) for 1 h, followed by overnight incubation at 4 °C and incubation with the secondary antibody (diluted 1:4000 in TBS-T containing 5% skim milk) for 1 h. Finally, protein bands were detected using Image Lab software (Bio-Rad, Hercules, CA, USA).

### 4.7. Quantitative Real-Time Polymerase Chain Reaction (qRT-PCR)

3T3-L1 cells (1 × 10^6^ cells/well) were scraped and centrifuged (14,000 rpm, 5 min, 20 °C). The resulting supernatant was removed and dissolved in 500 μL of NucleoZOL. Then, for total RNA extraction, a kit (NucleoSpin^®^ RNA Set for NucleoZOL, Macherey-Nagel) was used, and for cDNA synthesis, a ReverTra Ace qPCR RT kit was used, according to the manufacturer’s protocol. qRT-PCR was performed using the CFX384 Touch Real-Time PCR Detection System (Bio-Rad), with iQ™ SYBR Green Supermix and gene-specific primer sets. Table 1 shows the primer sequences used in this study.

### 4.8. Statistical Analysis

Triplicate data are expressed as the mean ± standard error of the mean. Differences between groups were evaluated using Student’s *t*-tests in GraphPad prism 8 (GraphPad Software, San Diego, CA, USA), and *p*-values < 0.05 were considered statistically significant.

## 5. Conclusions

**Figure 7:**
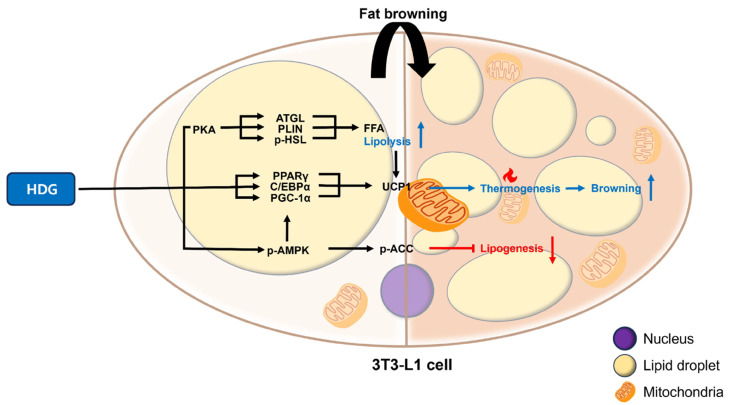
Fat browning process mediated by HDG. Stimulation: →, inhibition: ┫, increase: ↑, decrease: ↓.

We determined that HDG positively affected thermogenesis, significantly increasing thermogenesis-related factors. Figure 7 summarizes and simplifies this process. The increase and decrease in the expression levels of various targets suggest that the HDG compounds in H. helix have the potential to control obesity by enhancing thermogenesis and promoting fat browning. Our study has established a foundation at the in vitro level and serves as a cornerstone for conducting long-term investigations, such as on assessing the safety and efficacy of HDG treatment in both animal models and human subjects.

## Figures and Tables

**Table 1:** The primer sequence used for qRT-PCR.

Gene	Forward	Reverse
*Aco1*	ATCCAGACTTCCAACATFAG	AACCACATGATTTCTTCAGG
*Atgl*	TTCACCATCCGCTTGTTGGAG	AGATGGTCACCCAATTTCCTC
*Cd137*	GGTCTGTGCTTAAGACCGGG	TCTTAATAGCTGGTCCTCCCTC
*Cebpa*	AGGTGCTGGAGTTGACCAGT	CAGCCTAGAGATCCAGCGAC
*Cebpb*	ACGAGTACAAGATGCGGCG	TGAACAAGTTCCGCAGGGTG
*Cidea*	CGGGAATAGCCAGAGTCACC	TGTGCATCGGATGTCGTAGG
*Cited1*	AACCTTGGAGTGAAGGATCGC	GTAGGAGAGCCTATTGGAGATGT
*Cox4*	TGACGGCCTTGGACGG	CGATCAGCGTAAGTGGGGA
*Cpt1*	GTGTTGGAGGTGACAGACTT	CACTTTCTCTTTCCACAAGG
*Fgf21*	CGTCTGCCTCAGAAGGACTC	TCTACCATGCTCAGGGGGTC
*Hsl*	GCACTGTGACCTGCTTGGT	CTGGCACCCTCACTCCATA
*Nrf1*	GCTAATGGCCTGGTCCAGAT	CTGCGCTGTCCGATATCCTG
*Pgc1a*	ATGTGCAGCCAAGACTCTGTA	CGCTACACCACTTCAATCCAC
*Plin1*	GCAAGAAGAGCTGAGCAGAC	AATCTGCCCACGAGAAAGGA
*Ppara*	GAGAGGGCACACGCTAGGAA	GAACACCAATGTTCGGAGCC
*Pparg*	CAAGAATACCAAAGTGCGATCAA	GAGCTGGGTCTTTTCAGAATAATAAG
*Prdm16*	GATGGGAGATGCTGACGGAT	TGATCTGACACATGGCGAGG
*Tbx1*	AGCGAGGCGGAAGGGA	CCTGGTGACTGTGCTGAAGT
*Tfam*	ATGTGGAGCGTGCTAAAAGC	GGATAGCTACCCATGCTGGAA
*Tmem26*	CCATGGAAACCAGTATTGCAGC	ATTGGTGGCTCTGTGGGATG
*Ucp1*	CCTGCCTCTCTCGGAAACAA	GTAGCGGGGTTTGATCCCAT
*GAPDH*	TTGTTGCCATCAACGACCCC	GCCGTTGAATTTGCCGTGAG

## Data Availability

The data presented in this research are available on request from the corresponding author.
